# The role of flow experience and psychological commitment in recreation specialization and life satisfaction: evidence from Chinese marathon participants

**DOI:** 10.3389/fpsyg.2026.1753578

**Published:** 2026-02-27

**Authors:** Jiao Xu, Haibo Tian

**Affiliations:** 1 Department of Physical Education and Aesthetic Education, Hangzhou City University, Hangzhou, China; 2 Department of Physical Education, Shaoxing University, Shaoxing, China

**Keywords:** flow experience, life satisfaction, marathon, psychological commitment, recreation specialization

## Abstract

**Introduction:**

Earlier research has indicated an insufficient exploration of the way recreation specialization influences marathon runners’ life satisfaction. This study proposes a conceptual model designed to clarify the relationships between recreation specialization, flow experience, psychological commitment, and life satisfaction.

**Methods:**

Participants included 404 marathon runners randomly selected from the 2022 Shaoxing International Marathon Event in China. They completed self - report measures on recreation specialization, flow experience, psychological commitment, and life satisfaction. A series of mediation analyses using Amos 22.0 and JASP 0.16.1 were conducted for the study hypotheses.

**Results:**

The findings show that (a) recreation specialization has a direct and positive impact on life satisfaction; (b) psychological commitment completely mediates the influence of recreation specialization on life satisfaction, and (c) flow experience fully mediates the influence of recreation specialization on life satisfaction, with partially mediating through psychological commitment.

**Conclusion:**

These results add to the current literature by shedding light on how engaging in leisure sports can boost runners’ life satisfaction and uncovering the hidden mechanisms.

## 1 Introduction

The primary contradiction in contemporary China has shifted to the tension between people’s increasing demands for a better quality of life and an imbalanced or inadequate development situation (Lin, 2018). As outlined in “Healthy China 2030,” achieving a healthy and longer lifespan is a crucial indicator of national prosperity and rejuvenation, as well as a shared aspiration among all citizens. Therefore, improving individuals’ life satisfaction has become an important issue that Chinese scholars are increasingly focusing on. Recent research indicates that participation in leisure sports is closely linked with life satisfaction because it helps enhance physical fitness and psychological well-being (Kim et al., 2011; Sato et al., 2015; Yang et al., 2019). Marathon running, which is widely popular due to its social-psychological functions and leisure attributes, has been successfully held in over 100 cities across China and continues to attract more participants (Qiu et al., 2020; Zhang et al., 2020). Moreover, runners exhibit significant characteristics of recreation specialization throughout the entire process of marathon training and racing (Tian et al., 2022b). Previous studies have confirmed that marathon running can foster self-discipline, alleviate anxiety caused by status panic, increase social identity, and improve life satisfaction (Tian et al., 2020; Wang, 2019). Thus, this study holds both theoretical and practical significance for examining the role of flow experience and psychological commitment on the relationship between recreation specialization and life satisfaction among marathon runners.

Existing literature has established that recreation specialization has a significant positive impact on subjective well-being. However, the underlying mechanism through which recreation specialization influences individuals’ life satisfaction remains unclear (Sato et al., 2018; Tian et al., 2020). While previous studies have primarily focused on investigating the direct influence of recreation specialization on life satisfaction, they have overlooked the complexity and indirect nature of this relationship. It is worth noting that there may be several mediating factors involved in the role of recreation specialization in influencing life satisfaction. For example, Sato et al. (2016) suggested that behavioral loyalty directly affects life satisfaction, with psychological involvement acting as a mediator between behavioral loyalty and life satisfaction. Through the construction of a path model, Tian et al. (2022a) confirmed that loneliness serves as a mediator in the relationship between three dimensions of recreation specialization and successful aging. Therefore, it can be speculated that there exists a more intricate mechanism through which recreation specialization impacts life satisfaction. Previous studies (Tian et al., 2022b; Tian et al., 2022c) have separately examined the roles of the flow experience or psychological commitment as mediating variables to investigate the impact of recreation specialization on life satisfaction. They overlooked the consideration of incorporating psychological commitment and flow experience into the relationship between recreation specialization and life satisfaction simultaneously. Therefore, it is necessary to consider both psychological commitment and flow experience as mediating variables to examine the impact of recreation specialization on life satisfaction.

Above all, we posit that this study can offer a novel perspective in elucidating the role of recreation specialization on individuals’ life satisfaction. This investigation primarily focuses on two key issues: firstly, whether recreation specialization has a direct and positive impact on life satisfaction; secondly, whether flow experience and psychological commitment mediate the relationship between recreation specialization and life satisfaction. Consequently, this study will construct a hypothesis model to assess the relationship among recreation specialization, flow experience, psychological commitment, and life satisfaction within marathon runners.

## 2 Literature review and development of hypotheses

### 2.1 Recreation specialization and life satisfaction

Recreation specialization refers to a continuum of behavior ranging from general to specialized, reflecting the equipment and skills utilized in leisure activities, as well as the preference for activity sites or environments (Bryan, 1977). This concept has been widely employed to assess the diverse behavioral characteristics exhibited by participants engaging in outdoor leisure activities (Liu and Lou, 2019; Tian, 2021). Previous research has established a three-dimensional framework for recreation specialization, comprising behavior, cognition, and affect (Song et al., 2018). Marathon runners typically undergo a progressive journey from novice to expert and demonstrate notable attributes associated with recreation specialization such as sport commitment, knowledge acquisition, and skill development (Tian, 2021).

Life satisfaction reflects individuals’ cognitive evaluation of their overall quality of life and is considered an important indicator of subjective well-being (Diener et al., 1999). It is commonly defined as “a positive assessment of one’s entire life based on subjective criteria” (Pavot and Diener, 1993). Although previous literature has overlooked the examination of the impact of recreation specialization on life satisfaction, indirect and reliable evidence has been provided. A case study conducted on elderly cyclists explored the relationship between personality, recreation specialization, and subjective well-being, revealing that recreation specialization can significantly enhance individuals’ subjective well-being (Tian et al., 2023). Another study confirmed that the affect dimension of recreation specialization had a significant positive influence on life satisfaction among long-distance runners (Sato et al., 2018). Furthermore, serious participants reported experiencing higher levels of life satisfaction and health perception (Heo et al., 2013; Kim et al., 2011; Long and Wang, 2013). Additionally, demographic variables such as age and marital status also play a significant role in the relationship between leisure sports participation and individuals’ subjective well-being (Tian et al., 2020).

### 2.2 Flow experience and its mediating effect

Originating from Csikszentmihalyi’s (1975) study, flow experience has been frequently employed to elucidate individuals’ persistent engagement in certain activities devoid of material or economic gains. It denotes a psychological state wherein individuals immerse themselves in specific activities, strike a balance between challenges and skills, seamlessly integrate actions and consciousness, and derive intrinsic satisfaction (Csikszentmihalyi, 2002). Consequently, individuals develop disciplined exercise habits, accumulate extensive knowledge, skills, and experiences while exhibiting unwavering commitment towards engaging in leisure sports activities (Park et al., 2018). Recent research has demonstrated that leisure involvement significantly and positively impacts participants’ experiences (Cheng et al., 2016; Weng et al., 2020). Wu et al. (2013) further confirmed that individuals with higher levels of recreation specialization consistently encounter more frequent flow experience while recreation specialization moderates the influence of flow experience on addiction tendencies.

There exists a robust association between recreation specialization, flow experience, and life satisfaction. A higher level of recreation specialization indicates individuals’ persistent inclination towards their leisure choices (Bryan, 2000, 2001) and contributes to the attainment of enduring benefits such as self-image, self-satisfaction, and self-fulfillment (Tian et al., 2020). Previous research has suggested that recreation specialization significantly enhances individuals’ life satisfaction and subjective well-being (Heo et al., 2013; Kim et al., 2011; Tian et al., 2020). Drawing on data from an acrobatics show, a study identified flow experience as a crucial predictor and determinant of life satisfaction (Chen et al., 2010). Furthermore, recent findings have confirmed that flow experience plays a vital mediating role between demographics variables and life satisfaction among mountain bike tourists (Amatulli et al., 2021). In essence, it can be posited that recreation specialization enables individuals to attain heightened levels of flow experience while flow experience indirectly promotes the influence of recreation specialization on marathon runners’ life satisfaction.

### 2.3 Psychological commitment and its mediating effect

Commitment refers to the process in which people willing to give their time or energy to leisure activity in order to express their needs (Buchanan, 1985). Psychological commitment reflects individuals’ inclination to actively engage in preferred activities despite the availability of alternative options (Lee and Kyle, 2014; Pritchard et al., 1999). It is considered a crucial factor in understanding why people choose specific leisure activities or revisit particular destinations (Backman and Crompton, 1991; Pritchard et al., 1999). The level of psychological commitment naturally increases with the progression of individuals’ involvement in recreational pursuits (Stebbins, 2007). By constructing a structural equation model, Ridinger et al. (2012) discovered that both leisure involvement and negotiation efficiency significantly predict marathon runners’ perceived constraint. Additionally, Kyle et al. (2004) confirmed that higher levels of leisure involvement are associated with stronger levels of perceived constraint on leisure activities or destinations.

Previous studies have identified the crucial role of psychological commitment in mediating the relationship between leisure participation and sports experience. Cheng et al. (2016) confirmed a positive association between higher levels of leisure involvement and stronger perceptions of flow experience and psychological commitment, with psychological commitment found to mediate the link between leisure involvement and flow experience. Scholars have also established that dimensions of psychological commitment can indirectly influence individuals’ behavioral loyalty through their engagement in leisure activities (Iwasaki and Havitz, 2004). Furthermore, Sato et al. (2015, 2016, 2018) demonstrated that participating in marathon events leads to positive experiences and a sense of achievement, which contribute to enhancing runners’ life satisfaction. Tian et al. (2022b) provided evidence for the mediating effect of psychological commitment on the relationship between different dimensions of recreation specialization (i.e., behavior, cognition, and affect) and life satisfaction. Therefore, it is plausible that recreation specialization and flow experience positively impact runners’ psychological commitment; moreover, enhanced psychological commitment may facilitate improvements in life satisfaction while also serving as a mediator between recreation specialization and life satisfaction.

### 2.4 The hypotheses of this study

In general, the objectives of this study are twofold. The first is to evaluate the direct effects among recreation specialization and life satisfaction. The second is to investigate the mediating effects of flow experience and psychological commitment on the role of recreation specialization on life satisfaction. Based on the aforementioned discussion, the hypotheses of this study are as follows:

*H1:* Marathon runner’s recreation specialization positively influences their life satisfaction.

*H2:* The role of recreation specialization on life satisfaction is mediated by marathon runners’ flow experience.

*H3:* The role of recreation specialization on life satisfaction is mediated by marathon runners’ psychological commitment.

## 3 Methods

### 3.1 Participants and setting

The study comprised a total of 404 Chinese marathon runners. Within the sample, the majority of participants were male (269 or 66.6%). A significant proportion fell within the age range of 18 to 44 years old (368 or 91.1%). Most of the respondents possessed a college or university level education (284 or 70.3%), while a smaller fraction had completed high school or below (27 or 6.7%). The majority of runners were unmarried (251 or 62.1%), with over 30% being married individuals. Nearly half of the respondents reported an annual income exceeding US $3,001 (198 or 49%). Moreover, most participants were members of a running group (277or 68.6%) (See Table 1).

**Table 1:** Demographic characteristics of participants (*n* = 404).

Characteristics	Frequency (*n*)	Percentage (%)
Gender		
Male	269	66.6
Female	135	33.4
Age		
18–29	242	59.9
30–44	126	31.2
45–60	29	7.2
61 and above	7	1.7
Marital status		
Unmarried	251	62.1
Married	146	36.1
Divorced or widowed	7	1.7
Education		
High school or below	27	6.7
College or university	284	70.3
Postgraduate	93	23.0
Income (/year)		
US$3,000 and below	206	51.0
US$3,001–US$7,500	39	9.7
US$7,501–US$18,000	73	18.1
US$18,000 and above	86	21.3
Running group		
Joined	277	68.6
Not joined	127	31.4

### 3.2 Measurements

Recreation specialization was assessed using a modified version of a scale from previous studies (Iwasaki and Havitz, 2004; McIntyre and Pigram, 1992; Song et al., 2018), comprising nine items that encompassed three dimensions: behavior (three items), cognition (two items), and affect (four items). This measure aimed to evaluate the level of participation and engagement among marathon runners. Participants rated each item on a five-point Likert scale ranging from “strongly disagree” (scored 1) to “strongly agree” (scored 5). The recreation specialization statement used in this study was: “Running is important to me.” Higher scores indicated a greater degree of recreation specialization among marathon runners. Internal consistency analysis yielded Cronbach’s *α* coefficients ranging from 0.75 to 0.94 for the different dimensions of recreation specialization in this study. Confirmatory factor analysis results showed *χ*^2^ = 73.30, df = 24, RMSEA = 0.07, RMR = 0.04, CFI = 0.98, TLI = 0.96.

The Satisfaction with Life Scale (SWLS), developed by Diener et al. (1999), was employed to assess the overall quality of life among marathon runners. SWLS comprises five items, which are rated on a seven-point Likert scale ranging from “1” indicating “strongly disagree” to “7” representing “strongly agree.” The statement for life satisfaction was as follow: “In most ways my life is close to my ideal”. A higher total score indicates a greater level of life satisfaction among runners. In this study, the life satisfaction sub-scale demonstrated excellent internal consistency with a Cronbach’s *α* coefficient of 0.92.

Flow Short Scale (FSS), adapted from previous literature (Engeser and Rheinberg, 2008; Rheinberg et al., 2003), was employed to assess the flow state of marathon runners. The FSS comprises 10 items, encompassing absorption in activity (four items) and fluency of performance (six items). Participants rated these items on a seven-point Likert scale ranging from “1” indicating “not at all” to “7” representing “very much.” The statement for flow experience was as follow: “When running, I am totally absorbed in what I am doing”. Higher total scores indicate a stronger level of flow experience. The Cronbach’s *α* coefficients for flow experience dimensions were found to be 0.87 and 0.95, respectively. Confirmatory factor analysis yielded *χ*^2^ = 101.50, df = 34, RMSEA = 0.08, RMR = 0.05, CFI = 0.95, TLI = 0.93.

Psychological Commitment Scale (PCS), adapted from previous studies (Kyle et al., 2004; Pritchard et al., 1999), was utilized to assess the level of commitment among marathon participants. PCS comprised 10 items encompassing four dimensions: positional involvement (three items), resistance to change (three items), information complexity (two items), and volitional choice (two items). These items were rated on a seven-point Likert scale ranging from “strongly disagree” (score 1) to “strongly agree” (score 7). The statement for psychological commitment was as follows: “My preference to participation in running will not willingly change”. A higher total score indicates a stronger level of psychological commitment. The Cronbach’s α coefficient for the factors of psychological commitment ranged from 0.75 to 0.90. The confirmatory factor analysis yielded the following results: *χ*^2^ = 89.90, df = 29, RMSEA = 0.07, RMR = 0.06, CFI = 0.96, TLI = 0.94.

In accordance with previous research (Tian et al., 2020), a demographic variables questionnaire comprising six items, namely gender, age, marital status, education level, income, and membership of running group was employed by the researchers.

### 3.3 Procedures and data analysis

The data for this study were collected at the 2022 Shaoxing International Marathon Event, held on November 12 in Shaoxing, a city in Zhejiang province, China. This event has been officially recognized as a nationally influential event by the China Athletic Federation and consistently attracts over 15,000 runners annually since 2017. Based on the size of the event, the data collection process involved random sampling (i.e., every 25th runner) through Questionnaire Star, a web-based questionnaire platform deployed near the marathon finish area. The studies involving human participants were reviewed and approved by the Shaoxing University Ethics committee. The participants provided their written informed consent to participate in this study. A total of 432 questionnaires were initially collected; However, 28 samples were excluded from the data analysis according to Bryan’s (1977) definition of recreation specialization due to a low running frequency per week (e.g., running less than once per week). Consequently, a final sample size of 404 questionnaires was used to examine the study hypotheses.

The data in this study were analyzed using software of Amos 22.0 and JASP 0.16.1. Initially, confirmatory factor analysis was employed to assess the validity of all adapted scales. Subsequently, JASP was utilized to provide descriptive statistics and evaluate the internal consistency reliability of all variables, as well as test research Hypotheses 1 through Hypotheses 3.

## 4 Results

### 4.1 Common method variance test

To control for common method variance, this study employed Harman’s single—factor test to examine common method variance. The results indicated that the first unrotated factor accounted for only 35.60% of the total variance, which was less than 40% of the total explained variance. According to the suggestion of Tang and Wen (2020), this study does not have a serious common method bias.

### 4.2 Descriptive statistics, correlation analysis, and discriminant validity

The mean, standard deviation, and correlation coefficient of each variable are presented in Table 2. Marathon runners demonstrate higher levels of recreation specialization (M = 3.323; SD = 0.811), life satisfaction (M = 5.104; SD = 1.189), flow experience (M = 5.369; SD = 1.093), and psychological commitment (M = 5.369; SD = 1.164). The study findings reveal significant correlations among recreation specialization, life satisfaction, flow experience, and psychological commitment (*p* < 0.01), with correlation coefficients ranging from 0.489 to 0.851.

**Table 2:** Descriptive statistics, correlation, CR, and AVE of variables.

Constructs	M ± SD	CR	AVE	RS	LS	FE	PC
RS	3.323 ± 0.811	0.878	0.707	0.841			
LS	5.104 ± 1.189	0.912	0.912	0.489^**^	0.822		
FE	5.369 ± 1.093	0.783	0.783	0.619^**^	0.718^**^	0.804	
PC	5.369 ± 1.164	0.927	0.927	0.700^**^	0.675^**^	0.851^**^	0.874

(1)^**^p < 0.01; (2) RS, recreation specialization. LS, life satisfaction; FE, flow experience; PC, psychological commitment. The correlation coefficients are shown in the lower diagonals. The square roots of the AVE values are shown in boldface type.

Table 2 also shows the composite reliability, and square roots of the average variances extracted of recreation specialization, flow experience, psychological commitment, and life satisfaction. Previous research suggests that a recommended acceptable standard of an AVE value higher than 0.50 and a CR value higher than 0.70 (Fornell and Larcker, 1981). Overall, the composite reliability value ranged from 0.783 to 0.927; the average variances extracted ranged from 0.646 to 0.764. In addition, the square roots of the average variance extracted values of all the subscales were higher than the correlations with other constructs, demonstrating the high discriminant validity of all the subscales. Therefore, the constructs in the hypothesized model demonstrated acceptable reliability and validity.

### 4.3 Examining the direct effect between recreation specialization and life satisfaction

**Figure 1:**
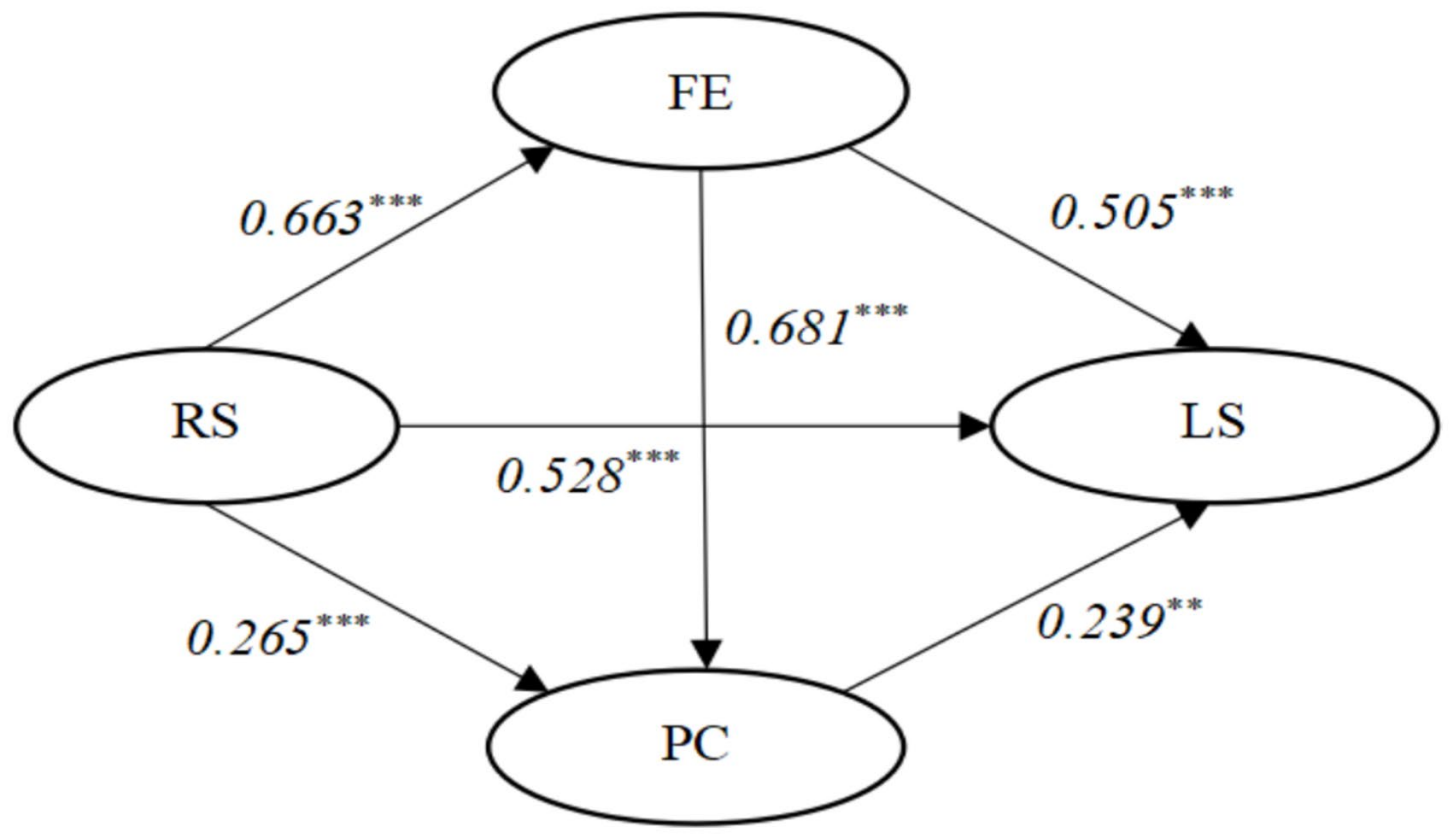
Standardized parameter estimate of conceptual model. CFI = 0.912; TLI: = 0.903; RMSEA = 0.035; ***p < 0.001, **p < 0.01; RS, recreation specialization; LS, life satisfaction; FE, flow experience; PC, psychological commitment.

The statistical software JASP 0.16.1 was utilized to analyze Hypotheses 1 through 3, with recreation specialization serving as the predictor variable, flow experience and psychological commitment as mediators, life satisfaction as the outcome variable, and demographic variables included as control variables. As shown in Table 3, the results indicate that a positive relationship was found between recreation specialization (*β* = 0.528, *p* < 0.001) and life satisfaction in the model without mediators, which supports H1 (see Figure 1).

**Table 3:** The regression analysis between recreation specialization and life satisfaction.

Hypothesis	β	SE	t	p
RS → LS	0.528	0.072	10.828	<0.001

RS, recreation specialization; LS, life satisfaction.

### 4.4 Mediating effect of flow experience and psychological commitment

The mediating effects were assessed and the 95% confidence intervals were computed, as presented in Table 4. The study findings provide confirmation that both flow experience and psychological commitment play a significant mediating role in the relationship between recreation specialization and life satisfaction, as indicated by the exclusion of zero from all confidence intervals. Therefore, H2 (a*b = 0.491, 95% CI = 0.271–0.659) and H3 (a*b = 0.093, 95% CI = 0.030–0.164) are supported, highlighting that flow experience partially explains its influence on life satisfaction through psychological commitment (See Table 4). However, the direct effect between recreation specialization and life satisfaction becomes non-significant (95% CI = −0.117–0.666) in the model with mediators. Therefore, it can be concluded that flow experience and psychological commitment fully mediate the impact of recreation specialization on life satisfaction.

**Table 4:** Bootstrap analysis on the testing mediation effect.

Hypothesis test	Effect value	Bootstrap SE	95% CI	
			Lower	Upper
Total effect	0.775	0.072	0.001	0.643
Direct effect	0.033	0.076	−0.117	0.666
Indirect effect	0.742	0.074	0.598	0.891
RS → FE → LS	0.491	0.099	0.271	0.659
RS → PC → LS	0.093	0.034	0.030	0.164
RS → FE → PC → LS	0.159	0.085	0.037	0.358

Using bootstrap method, 5,000 bootstrap re-sampling.

## 5 Discussion

A model was proposed to assess the impact of flow experience and psychological commitment on the relationship between recreation specialization and life satisfaction among Chinese marathon runners. This study contributes to existing literature in two key aspects: (a) examining the predictors of flow experience, psychological commitment, and life satisfaction; (b) investigating the influential pathways through which life satisfaction is affected by introducing flow experience and psychological commitment as mediating variables. The results of mediation analysis using JASP 0.16.1 provided support for all three hypotheses posited in this study.

In line with the findings of Heo et al. (2013) and Kim et al. (2011), the study results demonstrate a significant positive impact of recreation specialization on marathon runners’ life satisfaction in the model without mediators. Furthermore, this finding extends upon the research outcomes reported by Tian et al. (2023) and Sato et al. (2018). According to activity theory, both the frequency and emotional attachment to activities can effectively enhance individuals’ life satisfaction (Lemon et al., 1972). Tercan’s (2015) investigation into leisure participation, family assessment, and life satisfaction revealed that individuals who engage in leisure activities more frequently and for longer duration report higher levels of life satisfaction. Consistent with previous studies, marathon running holds great significance for individuals’ life satisfaction as it strengthens their physical well-being, alleviates life pressures, enhances social opportunities, and fosters a sense of achievement, belongingness, and identity (Shipway and Holloway, 2010). Therefore, these study results suggest that marathon runners should dedicate themselves to improving their level of recreation specialization in order to pursue higher levels of life satisfaction.

The bootstrap sampling method was employed to assess the mediating role of psychological commitment. The results indicate that psychological commitment mediates the impact of recreation specialization on life satisfaction, with a 95% confidence interval ranging from 0.030 to 0.164. The standardized path coefficient for recreation specialization to psychological commitment is 0.265 (*t* = 7.872, *p* < 0.001), and the standardized path coefficient for psychological commitment to life satisfaction is 0.239 (*t* = 3.322, *p* < 0.01). These findings suggest that recreation specialization significantly and positively influences psychological commitment, as does psychological commitment on life satisfaction. Previous literature has consistently demonstrated that engagement in leisure activities is associated with enhanced life satisfaction, with loneliness, social support, and flow experience serving as key mediators of the relationship between leisure participation and life satisfaction (Berger and Motl, 2000; Cheng et al., 2016). However, the specific impact mechanism of recreation specialization on life satisfaction remains unclear, particularly regarding the mediating role of psychological commitment. Building upon previous findings (Heo et al., 2013; Sato et al., 2018), this study confirms the mediating effect of psychological commitment based on data collected from marathon events.

Although previous studies have examined the impact of recreation specialization on life satisfaction, they have overlooked the examination of the mediating effect of flow experience. The study results demonstrate a significant positive impact of recreation specialization on flow experience; flow experience exhibits a significant positive effect on psychological commitment and life satisfaction. This study confirms that flow experience can mediate the role of recreation specialization in marathon runners’ life satisfaction, with its mediating effect partly exerted through psychological commitment. Consistent with Wu et al.’s (2013) and Wöran and Arnberger’s (2012) findings, individuals with higher levels of recreation specialization tend to experience stronger feelings of engagement. Furthermore, engaging in marathons significantly enhances both psychological commitment and life satisfaction (Chen et al., 2010; Wu et al., 2013). Previous research has established that flow experience plays a significant mediating role in leisure involvement’s influence on well-being (Weng et al., 2020; Wöran and Arnberger, 2012). However, existing literature has neglected to explore the indirect influence of flow experience on the relationship between recreation specialization and life satisfaction. This study verifies that flow experience acts as a mediator between recreation specialization and life satisfaction through two paths: an indirect effect generated by flow experience and a chain mediating effect involving both flow experience and psychological commitment. These findings contribute to further understanding the mechanism linking recreation specialization to life satisfaction in leisure sports activities while also suggesting that individuals should engage in leisure sports activities during their free time, select appropriate content and difficulty levels, pursue genuine emotions, thereby obtaining positive leisure experiences for long-lasting benefits towards improving their life satisfaction.

Our findings have implications for two perspectives. From a theoretical standpoint, our study extends previous research (Kim et al., 2011; Sato et al., 2018) to understand the relationship between recreation specialization and life satisfaction by examining the mediating effect of flow experience and psychological commitment. Furthermore, these findings contribute to understanding the predictors and influential pathways for life satisfaction. From a practical perspective, life satisfaction is positively influenced by recreation specialization progression. Individuals should pursue their original goals to enhance their level of recreation specialization by overcoming various constraints, acquiring knowledge and experience, and fostering social connections. For leisure sport organization organizers, it is highly recommended to adopt comprehensive and targeted strategies—such as organizing high-quality and well-structured marathon events, actively promoting the dissemination of professional marathon skills and knowledge through various channels, and providing thorough, participant-centered event services—to effectively enhance people’s overall life satisfaction and well-being. In addition to policy formulation, it is also crucial for government departments to collaborate with local communities and organizations. By establishing partnerships, they can develop programs that are not only in line with public health goals but also resonate with the cultural and social fabric of the community. Such initiatives could encompass wellness campaigns, recreational activities, and educational workshops that encourage healthier lifestyles while cultivating a sense of belonging and engagement among participants. Through these efforts, the overall well - being of individuals can be further supported, contributing to a more holistic approach to enhancing life satisfaction.

Although this study offers valuable insights into the relationship between recreation specialization, psychological commitment, flow experience, and life satisfaction in a leisure context, there are several limitations that need to be addressed. Firstly, this study only collected the data from Shaoxing Marathon events to explore the influence of recreation specialization on life satisfaction. However, it is important to note that the generalizability of these findings may be subject to some bias in other cultural and sports contexts. Future research should evaluate these findings in other types of sports activities and a broader cultural background. Secondly, the recreation specialization and life satisfaction of marathon runners were severely impacted against the backdrop of the COVID-19 pandemic. Therefore, the results should be re-examined after overcoming the COVID-19 pandemic. Thirdly, the sampling approach implemented in this study may introduce selection bias, such as in the case of runners with more available time or lower post - race fatigue. Future research can reduce this impact by increasing the sample size or adopting a more scientific sampling design. Lastly, Cross-sectional studies inevitably have certain limitations, such as the inability to determine causal relationships between variables, the difficulty in tracking the development and changes of individuals, and susceptibility to various biases. These limitations mainly stem from the characteristics of only collecting data at a single point in time and cannot capture dynamic trends. Therefore, by conducting longitudinal studies, it can deeply analyze the changing trajectories of each variable in the research model and the dynamic mechanisms of their interactions, thereby more comprehensively and accurately revealing the intrinsic connections between variables.

## 6 Conclusion

By employing a structural equation model, this study has examined the influence of recreation specialization on marathon runners’ life satisfaction, with flow experience and psychological commitment serving as mediators. The results revealed that recreation specialization had a direct and positive impact on life satisfaction in the model without mediators. Furthermore, flow experience and psychological commitment mediated the relationship between recreation specialization and life satisfaction, with flow experience partially mediating through psychological commitment. These findings contribute to our understanding of the role of recreation specialization in marathon runners’ life satisfaction. Moreover, they offer theoretical insights for individuals seeking to enhance their life satisfaction through leisure sports activities by cultivating self-discipline exercise habits, improving relevant knowledge and skills, selecting appropriate activity content or difficulty levels, enhancing flow experience, and fostering psychological commitment.

## Data Availability

The original contributions presented in the study are included in the article/supplementary material, further inquiries can be directed to the corresponding author.

## References

[ref1] Amatulli C. Peluso A. M. Sestino A. Petruzzellis L. Guido G. (2021). The role of psychological flow in adventure tourism: sociodemographic antecedents and consequences on word-of-mouth and life satisfaction. J. Sport Tour. 25, 353–369. doi: 10.1080/14775085.2021.1994448

[ref2] Backman S. J. Crompton J. L. (1991). Differentiating between high, spurious, latent, and low loyalty participants in two leisure activities. J. Park. Recreat. Adm. 9, 1–17.

[ref3] Berger B. G. Motl R. W. (2000). Exercise and mood: a selective review and synthesis of research employing the profile of mood states. J. Appl. Sport Psychol. 12, 69–92. doi: 10.1080/10413200008404214

[ref4] Bryan H. (1977). Leisure value systems and recreational specialization: the case of trout fishermen. J. Leis. Res. 9, 174–187. doi: 10.1080/00222216.1977.11970328

[ref5] Bryan H. (2000). Recreation specialization revisited. J. Leis. Res. 32, 18–21. doi: 10.1080/00222216.2000.11949879

[ref6] Bryan H. (2001). Reply to David Scott and C. Scott Shafer, "recreational specialization: a critical look at the construct". J. Leis. Res. 33, 344–347. doi: 10.1080/00222216.2001.11949945

[ref7] Buchanan T. (1985). Commitment and leisure behavior: a theoretical perspective. Leis. Sci. 7, 401–420. doi: 10.1080/01490408509512133

[ref8] Chen L. H. Ye Y. C. Chen M. Y. Tung I. W. (2010). Alegría! Flow in leisure and life satisfaction: the mediating role of event satisfaction using data from an acrobatics show. Soc. Indic. Res. 99, 301–313. doi: 10.1007/s11205-010-9581-z

[ref9] Cheng T. M. Hung S. H. Chen M. T. (2016). The influence of leisure involvement on flow experience during hiking activity: using psychological commitment as a mediate variable. Asia Pac. J. Tour. Res. 21, 1–19. doi: 10.1080/10941665.2014.1002507

[ref10] Csikszentmihalyi M. (1975). Beyond boredom and anxiety. Hoboken: Jossey-Bass.

[ref11] Csikszentmihalyi M. (2002). Flow: the psychology of optimal experience. New York, NY: Harper & Row.

[ref12] Diener E. Suh E. M. Lucas R. E. Smith H. L. (1999). Subjective weil-being:three decades of progress. Psychol. Bull. 125, 276–302. doi: 10.1037/0033-2909.125.2.276

[ref13] Engeser S. Rheinberg F. (2008). Flow, performance and moderators of challenge-skill balance. Motiv. Emotion. 32, 158–172.

[ref14] Fornell C. Larcker D. F. (1981). Evaluating structural equation models with unobservable variables and measurement error. J. Mark. Res. 18, 39–50. doi: 10.2307/3151312

[ref15] Heo J. Stebbins R. A. Kim J. Lee I. (2013). Serious leisure, life satisfaction, and health of older adults. Leis. Sci. 35, 16–32. doi: 10.1080/01490400.2013.739871

[ref16] Iwasaki Y. Havitz M. E. (2004). Examining relationships between leisure involvement, psychological commitment and loyalty to a recreation agency. J. Leis. Res. 36, 45–72. doi: 10.1080/00222216.2004.11950010

[ref17] Kim J. Dattilo J. Heo J. (2011). Taekwondo participation as serious leisure for life satisfaction and health. J. Leis. Res. 43, 545–559. doi: 10.1080/00222216.2011.11950249

[ref18] Kyle G. Graefe A. Manning R. Bacon J. (2004). Predictors of behavioral loyalty among hikers along the Appalachian Trail. Leis. Sci. 26, 99–118. doi: 10.1080/01490400490272675

[ref19] Lee J. J. Kyle G. T. (2014). Segmenting festival visitors using psychological commitment. J. Travel Res. 53, 656–669. doi: 10.1177/0047287513513168

[ref20] Lemon B. W. Bengtson V. L. Peterson J. A. (1972). An exploration of the activity theory of aging: activity types and life satisfaction among in-movers to a retirement community. J. Gerontol. 27, 511–523. doi: 10.1093/geronj/27.4.511, 5075497

[ref21] Lin Z. M. (2018). Correctly understand the transformation of China’s major social contradictions. Beijing: people’s daily, A7.

[ref22] Liu S. Lou J. J. (2019). Can we play seriously? A literature review of recreation specialization. Tour. Hosp. Prospect. 3, 64–82. doi: 10.12054/lydk.bisu.116

[ref23] Long J. Z. Wang S. (2013). Serious leisure and happy life: a localization study based on Chinese senior group. Tour. Trib. 28, 77–85.

[ref24] McIntyre N. Pigram J. J. (1992). Recreation specialization reexamined: the case of vehicle-based campers. Leis. Sci. 14, 3–15. doi: 10.1080/01490409209513153

[ref25] Park S. H. Hsieh C. Miller J. C. (2018). Moderating effects of recreation specialization on the quality-value-loyalty chain: a case of the Taroko gorge Marathon. Int. J. Tour. Sci. 18, 29–42. doi: 10.1080/15980634.2018.1438102

[ref26] Pavot W. Diener E. (1993). The satisfaction with life scale. Psychol. Assess. 2, 164–172. doi: 10.1037/1040-3590.5.2.164

[ref27] Pritchard M. P. Havitz M. E. Howard D. R. (1999). Analyzing the commitment-loyalty link in service contexts. J. Acad. Market Sci. 27, 333–348. doi: 10.1177/0092070399273004

[ref9001] Qiu Y. Tian H. Lin Zhou W. (2020). Serious leisure qualities and participation behaviors of Chinese marathon runners. Int. Rev. Sociol. Spor. 55, 523–543. doi: 10.3389/fpsyg.2025.1653610

[ref28] Rheinberg F. Vollmeyer R. Engeser S. (2003). “The assessment of flow experience” in Diagnosis of motivation and self-concept. eds. Stiensmeier-Pelster J. Rheinberg F. (Göttingen: Hogrefe), 261–279.

[ref29] Ridinger L. L. Funk D. C. Jordan J. S. Kaplanidou K. (2012). Marathons for the masses: exploring the role of negotiation-efficacy and involvement on running commitment. J. Leis. Res. 44, 155–178. doi: 10.1080/01419870.2012.668205

[ref30] Sato M. Jordan J. S. Funk D. C. (2015). Distance running events and life satisfaction: a longitudinal study. J. Sport Manag. 29, 347–361. doi: 10.1123/JSM.2013-0164

[ref31] Sato M. Jordan J. S. Funk D. C. (2016). A distance-running event and life satisfaction: the mediating roles of involvement. Sport. Manag. Rev. 19, 536–549. doi: 10.1080/00222216.2018.1425051

[ref32] Sato M. Jordan J. S. Funk D. C. Sachs M. L. (2018). Running involvement and life satisfaction: the role of personality. J. Leisure Res. 49, 28–45. doi: 10.1080/00222216.2018.1425051

[ref33] Shipway R. Holloway I. (2010). Running free: embracing a healthy lifestyle through distance running. Perspect. Public Health 130, 270–276. doi: 10.1177/1757913910379191, 21213563

[ref34] Song H. Graefe A. Kim K. Park C. (2018). Identification and prediction of latent classes of hikers based on specialization and place attachment. Sustainability 10:1163. doi: 10.3390/su10041163

[ref35] Stebbins R. A. (2007). Serious leisure: a perspective for our time. Piscataway, NJ: Transaction Publishers.

[ref36] Tang D. Wen Z. (2020). Statistical approaches for testing common method bias: problems and suggestions. J. Psychol. Sci. 43, 215–223. doi: 10.16719/j.cnki.1671-6981.20200130

[ref37] Tercan E. (2015). An examination of leisure participation, family assessment and life satisfaction in university students. Procedia. Soc. Behav. Sci. 186, 58–63. doi: 10.1016/j.sbspro.2015.04.123

[ref38] Tian H. (2021). The performance characteristics of leisure sport specialization (dissertation thesis. Hangzhou: Zhejiang University.

[ref39] Tian H. Qiu Y. Lin Y. Zhou W. (2023). Personality and subjective well-being among elder adults: examining the mediating role of cycling specialization. Leis. Sci. 45, 117–134. doi: 10.1080/01490400.2020.1790063

[ref40] Tian H. B. Qiu Y. J. Lin Y. Q. Zhou W. T. Fan C. Y. (2020). The role of leisure satisfaction in serious leisure and subjective well-being: evidence from Chinese marathon runners. Front. Psychol. 11, 1–10. doi: 10.3389/fpsyg.2020.581908 33329237 PMC7720892

[ref41] Tian H. Zhou W. Qiu Y. (2022b). The mediating role of psychological commitment between recreation specialization and life satisfaction: evidence from Xiamen marathon runners. Front. Psychol. 13, 1–11. doi: 10.3389/fpsyg.2022.1006289, 36425842 PMC9679789

[ref42] Tian H. Zhou W. Qiu Y. Shang Y. (2022a). The impact of cycling specialization on successful aging and the mediating role of loneliness. Int. J. Environ. Res. Public Health 19:19. doi: 10.3390/ijerph19010019, 35010276 PMC8750278

[ref43] Tian H. Zhou W. Qiu Y. Zou Z. (2022c). The role of recreation specialization and self-efficacy on life satisfaction: the mediating effect of flow experience. Int. J. Environ. Res. Public Health 19:3243. doi: 10.3390/ijerph19063243, 35328931 PMC8950726

[ref44] Wang J. (2019). Fashion and identity: social and cultural analysis of young middle class participating in marathon. Chin. Youth Soc. Sci. 38, 109–116. doi: 10.16034/j.cnki.10-1318/c.2019.02.030

[ref45] Weng L. Lei Y. Pan X. (2020). Effects of leisure involvement on the well-being of elderly people: mediation effects of self-efficacy and flow experience. J. Shanghai Univ. Sport 44, 87–94. doi: 10.16099/j.sus.2020.09.009

[ref46] Wöran B. Arnberger A. (2012). Exploring relationships between recreation specialization, restorative environments and mountain hikers’ flow experience. Leis. Sci. 34, 95–114. doi: 10.1080/01490400.2012.652502

[ref47] Wu T. E. Scott D. Yang C. (2013). Advanced or addicted? Exploring the relationship of recreation specialization to flow experiences and online game addiction. Leis. Sci. 35, 203–217. doi: 10.1080/01490400.2013.780497

[ref48] Yang H. T. Kim J. Heo J. (2019). Serious leisure profiles and well-being of older Korean adults. Leis. Stud. 38, 88–97. doi: 10.1080/02614367.2018.1499797

[ref49] Zhang H. Luo J. Y. Bai X. J. Yang X. F. (2020). The relations between the marathon culture and the urban residents’ happiness index: based on the survey of local event participants. Chin. Sport Sci. Technol. 56, 32–39. doi: 10.16470/j.csst.2019237

